# MXene-Derived Oxide Nanoheterostructures for Photocatalytic Sulfamethoxazole Degradation

**DOI:** 10.1021/acsanm.4c02523

**Published:** 2024-07-18

**Authors:** Shalu Atri, Elham Loni, Frantisek Zazimal, Karol Hensel, Maria Caplovicova, Gustav Plesch, Xin Lu, Rajamani Nagarajan, Michael Naguib, Olivier Monfort

**Affiliations:** † Department of Inorganic Chemistry, Faculty of Natural Sciences, Comenius University, Ilkovicova 6, Mlynska dolina, 84215 Bratislava, Slovakia; ‡ Department of Physics and Engineering Physics, Tulane University, New Orleans, Louisiana 70118, United States; § Department of Plasma Physics and Technology, Faculty of Science Masaryk University, Masaryk University, Kotlarska 267/2, 611 37 Brno, Czechia; ∥ Division of Environmental Physics, Faculty of Mathematics Physics and Informatics, Comenius University, Mlynska dolina, 84248 Bratislava, Slovakia; ⊥ STU Center for Nanodiagnostics, Faculty of Materials Science and Technology in Trnava, Slovak Technical University, Vazovova 5, 81243 Bratislava, Slovakia; # Materials Chemistry Group, Department of Chemistry, University of Delhi, Delhi 110007, India; ∇ Department of Chemistry, Tulane University, New Orleans, Louisiana 70118, United States

**Keywords:** binary MXene, photocatalyst, sulfamethoxazole, wastewater treatment, TiNbO_*x*_

## Abstract

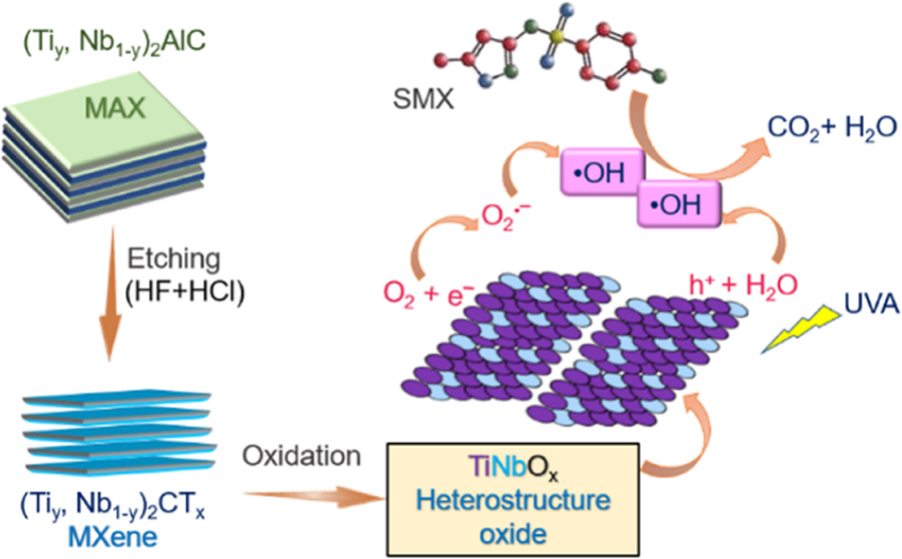

Herein, we report for the first time the use of ternary oxide nanoheterostructure photocatalysts derived from (Nb_*y*_, Ti_1–*y*_)_2_CT_*x*_ MXene in the treatment of water. Three different compositions of binary MXenes, viz., (Ti_0.75_Nb_0.25_)_2_CT_*x*_, (Ti_0.5_Nb_0.5_)_2_CT_*x*_, and (Ti_0.25_Nb_0.75_)_2_CT_*x*_ (with T_*x*_ = OH, F, and Cl), were used as single-source precursor to produce TiNbO_*x*_-3:1, TiNbO_*x*_-1:1, and TiNbO_*x*_-1:3 by controlled-atmosphere thermal oxidation. Phase identification and Le Bail refinements confirmed the presence of a mixture of rutile TiO_2_ and monoclinic Ti_2_Nb_10_O_29_. Morphological investigations through scanning and transmission electron microscopies revealed the retention of layered nanostructures from the MXene precursors and the fusion of TiO_2_ and Ti_2_Nb_10_O_29_ nanoparticles in forming nanosheets. Among the three oxide nanoheterostructures, TiNbO_*x*_-3:1 exhibited the best photocatalytic performance by the removal of 83% of sulfamethoxazole (SMX) after 2 h of reaction. Such a result is explained by a complex influence of structural, morphological, and electronic properties since TiNbO_*x*_-3:1 consisted of small-sized crystallites (40–70 nm) and possessed a higher surface area. The suggested electronic band structure is a type-II heterojunction, where the recombination of electrons and holes is minimized during photocatalytic reactions. The photocatalytic degradation of SMX was promoted by the attack of ^•^OH, as evidenced by the detection of 2.2 μM ^•^OH, using coumarin as a probe. This study highlights the potential application of MXene-derived oxide nanoheterostructures in wastewater treatment.

## Introduction

Heterogeneous photocatalysis is one of the most investigated solar conversion processes,^1^ and it has the potential to fulfill the global need for sustainability. It is a cost-effective and environment-friendly technology for wastewater treatment and production of clean energy, for example, to produce hydrogen from water splitting,^2,3^ CO_2_ conversion into valuable chemicals,^4,5^ and degradation of organic pollutants.^6,7^ Numerous photocatalysts, such as transition metal oxides, sulfides, nitrides, and many more, have been explored for water or air treatment.^2−8^ Concerning water treatment, the continuous stress of harmful pharmaceutical active compounds (PACs) on the water bodies is an environmental problem of high concern for human and animal life.^9^ Sulfamethoxazole (SMX) is widely used as a sulfonamide antibiotic and is one of the most frequently detected PACs in wastewater from pharmaceutical companies and municipalities.^10^ Due to the high chemical stability and poor biodegradability of SMX, conventional methods are inefficient in eliminating SMX.

Titanium dioxide (TiO_2_) has been proven as an efficient photocatalyst, but it is only active under the UVA spectrum due to the mismatch between its energy band gap (*E*_g_) and the solar spectrum, which contains only about 5% of UVA irradiation.^11^ This issue has given rise to thousands of research articles dealing with dozens of different approaches, including morphological and composition modifications to enhance the photocatalytic efficiency of TiO_2_.^12^ One of these approaches is to design nanoheterostructure materials, as they offer higher light-harvesting properties and effectiveness in retarding the recombination of charge carriers.^13,14^ This phenomenon occurs due to unequal Fermi levels of the two components that allow the transport of electrons at the coupling interface.^15^ Therefore, the separation and migration of charge carriers can be enhanced.^16^

MXenes with the M_*n*+1_X*_n_*T_*x*_ formula (M: early transition metal, X: C and/or N, T_*x*_: functional groups, *n* = 1–4)^17^ are two-dimensional (2D) multilayered materials that recently emerged as cocatalysts for photocatalytic applications due to their unique structural and electronic properties.^18,19^ As photocatalysts, the formed MXene-derived transition metal oxides (MO_*x*_) can be highly efficient nanomaterials for photocatalytic applications.^20,21^ MXene-derived MO_*x*_ are obtained by partial or complete oxidation of MXene (Ti_3_C_2_T_*x*_ and Ti_2_CT_*x*_) depending on oxidation conditions, and they exhibit superior properties compared to MO_*x*_/MXene composites.^22,23^ Recent advancements in MXene-based photocatalysts highlight their growing significance in environmental and energy challenges, and ongoing research aims to overcome the current limitations and scale up their production for practical applications.^24−27^ Reported studies pointed out that oxygen-containing functional groups, especially, facilitate the formation of MO_*x*_ phases.^28^ It has been reported that TiO_2_/amorphous carbon sheets can be derived by using flash-heating multilayered Ti_3_C_2_T_*x*_ in air.^29^ The use of MXene as the precursor to produce the oxide material is promising since it provides a unique 2D structure, morphology, and electronic properties that cannot be achieved by employing other synthetic routes.^30,31^ Such properties are the key factors in tuning and enhancing the photocatalytic behavior of a material.^32,33^

**Figure 1:**
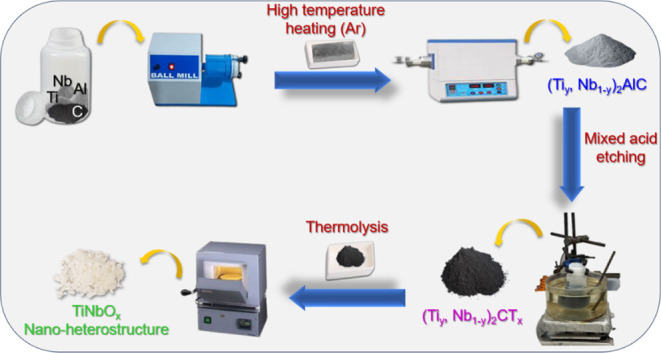
Schematic representation of the preparation of TiNbO_*x*_.

Taking cognizance of other reported studies, the current work is focused on designing a new type of nanoheterostructure oxide photocatalyst derived from (Nb_*y*_Ti_1–*y*_)_2_CT_*x*_ MXene as a precursor (Figure 1) since they can exhibit superior physicochemical properties than other Ti and Nb oxide composites.^34,35^ Indeed, retaining the layered structure of MXene is an innovative direction to obtain a more efficient photocatalyst for the degradation of organic pollutants in water. In addition, Nb-substituted binary MXene was used as the precursor for the first time. The selection of Nb was based on the possibility to tune the electronic properties of the resulting oxide nanoheterostructure photocatalyst.^36,37^ Such a methodology has not yet been tested yet. Three oxide nanoheterostructures (TiNbO_*x*_) have been prepared, i.e., (i) one with an equal nominal amount of Ti and Nb, (ii) one with a predominant Ti oxide phase, and (iii) one with a predominant Nb oxide phase. A relationship between the photocatalytic activity of such innovative materials and their corresponding properties, including the crystalline phase, composition, morphology, surface chemistry, and electronic band structure, has also been presented.

## Experimental Section

This work integrates the preparation of (Ti, Nb)_2_AlC (MAX phases) and their corresponding MXene and oxide nanoheterostructures. For MAX synthesis, the used reactant powders titanium (Ti, Alfa Aesar, 99.5% metal basis, -325 mesh), niobium (Nb, Thermo scientific, 99.8% metal basis, -325 mesh), aluminum (Al, Thermo scientific, 99.5% metal basis, -325 mesh) and carbon (Alfa Aesar, 99% C, 0.2% ash, graphite powder, APS-7-11 micron) were of analytical standard.

### Synthesis of TiNbO_*x*_

First, the MAX phase powders were prepared by mixing Ti, Nb, Al, and C powders in a stochiometric molar ratio to prepare (Ti_0.75_Nb_0.25_)_2_AlC, (Ti_0.5_Nb_0.5_)_2_AlC, and (Ti_0.25_Nb_0.75_)_2_AlC. An excess of 0.2 Al was used to compensate for any evaporation during heating. The mixture was subjected to mixing at 56 rpm for 3 h in the presence of 20 yttria-stabilized zirconia balls of 10 mm diameter in a Turbula T2F mixer. Then, the powders were transferred to an alumina boat, inserted inside a tube furnace, and heated at 1500 °C for 3 h with a heating rate of 10 °C min^–1^ under a continuous flow of argon (Ar) of 0.4 mL min^–1^. The obtained powders were referred to as MAX phase powders.

For the synthesis of (Ti_0.25_Nb_0.75_)_2_CT_*x*_ MXene, 1.0 g of (Ti_0.25_Nb_0.75_)_2_AlC powder was slowly added to 10 mL of aqueous hydrofluoric acid solution (HF, Thermo Scientific, ACS Reagent, 48–51% solution in water) placed in an ice bath to avoid overheating due to exothermic reactions. The solution mixture was stirred in an oil bath at 40 °C for 72 h. For (Ti_0.75_Nb_0.25_)_2_CT_*x*_ and (Ti_0.5_Nb_0.5_)_2_CT_*x*_, a similar approach was followed but using a mixture of HF (48–51%) and hydrochloric acid (HCl, Thermo Scientific, for analysis, 37% solution) in a volume ratio of 60:40 at continuous stirring at 25 °C for 30 h. After being etched, the powders, acids, and DI water were transferred to centrifuge tubes and centrifuged at 3500 rpm for 2 min. The supernatant acid was removed, and the washing procedure was repeated to reach pH 7. The settled powders in the centrifuging tubes were extracted using DI water, and the dispersion was vacuum-filtered and dried at room temperature.

The oxidized MXenes (TiNbO_*x*_) were prepared by heat treatment of the corresponding 1.0 g of MXene in an alumina crucible with dimensions 100 mm × 40 mm × 40 mm in a muffle furnace at 900 °C for 1 h in air with a heating rate of 5 °C min^–1^. The obtained white powders were characterized and utilized to be investigated for photocatalytic applications.

### Photocatalytic Setups and Related Analyses

The prepared TiNbO_*x*_ powders were tested for the photocatalytic degradation of sulfamethoxazole (SMX, Merck, VETRANAL, analytical standard) under UVA light (1.5 mW cm^–2^ in the wavelength range of 335–380 nm) in batch mode. A double-walled cylindrical pyrex container of volume 100 mL capacity was used and thermostated at 20 °C to minimize thermal effects. In each experiment, 0.2 g L^–1^ photocatalysts were added to 50 mL of 50 μM SMX solution in deionized water; the reaction mixture was stirred in the dark for 20 min to attain the adsorption–desorption equilibrium before irradiation at room temperature. The degradation extents were determined at fixed time intervals by sampling out 500 μL from the solution that was filtered using a poly(tetrafluoroethylene) (PTFE) microfilter with a pore size of 0.45 μm and quenched into 100 μL of methanol to stop the degradation reaction. The collected samples were analyzed by high-performance liquid chromatography (HPLC, Merck AS-2000 L-6200A L-4250) equipped with a C18 column (Hypersil Gold, 5 μm, 150 mm × 4.6 mm; Thermo Fisher Scientific). The mobile phase was a mixture of MeOH:H_2_O (50:50) at a flow rate of 1 mL min^–1^ in isocratic mode. The detection wavelength was set at 268 nm.

Reactive species were identified indirectly by adding a scavenger to the reactive mixture. The *tert*-butanol (10 mM) scavenger was used to quench hydroxyl radicals (^•^OH),^38^ and their quantification was performed using a fluorescence spectrophotometer (Shimadzu RF-6000) using coumarin as a probe molecule since their reaction forms 7-hydroxycoumarin (with a yield of 4.6%), which is fluorescent (λ_ex_ = 325 nm; λ_em_ = 425 nm).^39^

### Characterizations

The crystalline phase identification and purity were carried out by X-ray diffraction (XRD) using a Cu Kα X-ray diffractometer (Rigaku D/Max-2200) at a 2θ step size of 0.02° and a sweep rate of 1° min^–1^. Crystal lattice details were gathered using Le Bail refinements and general structure analysis system (GSAS) software.^40,41^ Raman spectroscopy measurements were conducted using a home-built setup to obtain information about the chemical structure and molecular interactions. The measurements were performed in a backscattering configuration excited with a solid-state green laser (λ = 532 nm). To reach the ultralow frequency Raman shift of ∼10 cm^–1^, we used the volume Bragg grating filters to block the laser line. The backscattered signal was collected through a 100× objective and dispersed by an 1800 g/mm grating before the liquid nitrogen-cooled charge-coupled device (Princeton Instruments, PyLoN 1340 × 400 pixels charge-coupled device (CCD)). Fourier transform infrared spectroscopy (FTIR, Vertex 70v, Bruker) with a diamond attenuated total reflectance (ATR) accessory was complementarily used for chemical analysis. The infrared spectra of all samples were recorded in the mid-IR range of 4000–400 cm^–1^ with a spectral resolution of 4 cm^–1^. The recorded spectra are the mean of 32 scans. Before spectral acquisition, a background spectrum (air) was measured with the same parameters. OPUS software was used for background correction and transformation to the resulting absorbance spectra of samples.

Scanning electron microscopy (SEM, Hitachi S-4800, at 20 kV) along with energy-dispersive X-ray spectroscopy (EDS, ULTIM MAX 170, Oxford, at 20 kV) and transmission electron microscopy (double-corrected TEM, JEOL JEM ARM 200 cF with a cold field emission gun) including high-resolution transmission electron microscopy (HRTEM), bright-field scanning transmission electron microscopy (BF STEM), secondary electron scanning transmission electron microscopy (SEI STEM), and selected area electron diffraction (SAED) patterns were performed to provide further details on the structure and morphology of the samples. A large-angle JEOL JED-2300T CENTURIO SDD (silicon drift) detector with a solid angle of up to 0.98 sr and a detection area of 100 mm^2^ was used for energy-dispersive X-ray spectroscopy (EDX) analysis. For the TEM study, powder samples were dispersed in ethanol, and suspensions were sonicated for 10 min and dropped on a Cu grid covered with a holey carbon film. After being dried in the air, they were examined using a TEM, working at 200 kV.

The surface chemistry was studied by X-ray photoelectron spectroscopy (XPS) using an AXIS supra spectrometer. An X-ray Al Kα source (15 mA and 15 kV) generating a monochromatic beam of photons of 1486.6 eV energy was used. The charging of the samples was compensated for using an automatic electron-flood gun system. The wide and core-level spectra were acquired at pass energies of 160 and 20 eV, respectively. The measurements were performed at 2 spots on the sample. The pressure in the working chamber was in the order of 10^–7^ Pa. The measured data were analyzed by CasaXPS software.^42^ The spectra were referenced using the C 1s signal at 284.8 eV, corresponding to C–C bonds. The background signal was subtracted by the Shirley algorithm. The synthetic components of the C 1s spectra were assigned and fitted based on parameters proposed by Biesinger et al.^43^ The Ti 2p spectra were fitted based on the parameters discussed by Biesinger et al.^44^ The components of the Nb 3d spectra were assigned based on the NIST database.^45^ The fit of the Nb 3d spectra was performed using symmetrical mixed Gaussian–Lorentzian components.

The surface area measurements were recorded using BET (the Brunauer–Emmet–Teller method, Sorptomatic 1990 SERIES, Thermo Quest CE Instruments, Italy) in the relative pressure range *p*/*p*_0_ = 0.05–0.25. Adsorption–desorption isotherms were measured at *p*/*p*_0_ = 0–1, with the low-temperature adsorption method of N_2_ at its boiling point of 77.7 K from vacuum to atmospheric pressure. The optical properties of the samples were measured by ultraviolet–visible (UV–vis) diffuse reflectance spectroscopy (DRS) using a PerkinElmer Lambda-35 spectrophotometer with a 50 mm integrating sphere and using BaSO_4_ as an external reference. The measured reflectance spectra were transformed by the Kubelka–Munk algorithm, and the Tauc plot was applied to determine the band gap energy (*E*_g_) based on the work.^46^

## Results and Discussion

**Figure 2:**
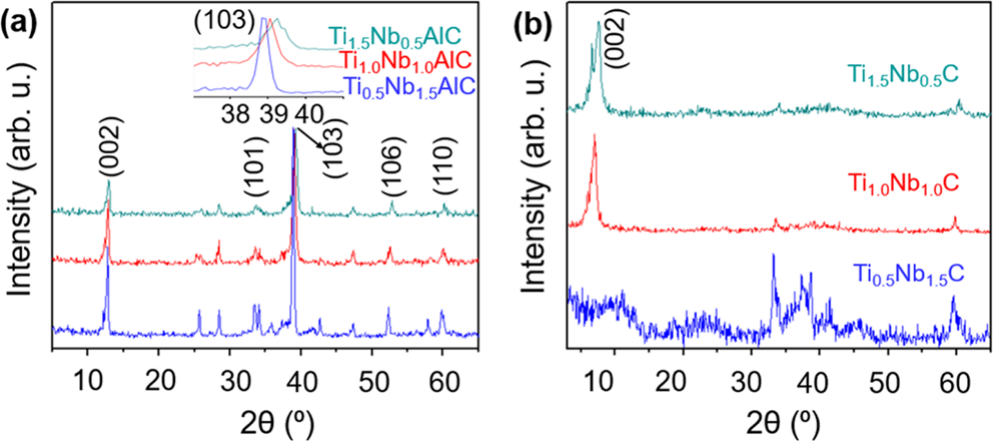
XRD patterns of Nb-substituted binary (a) MAX phases and (b) MXene powders. The inset in panel (a) shows the zoomed-in region from 2θ of 37 to 41°.

The formation of the three different MAX phase powders, i.e., (Ti_0.75_Nb_0.25_)_2_AlC, (Ti_0.50_Nb_0.5_)_2_AlC, and (Ti_0.25_Nb_0.75_)_2_AlC, was confirmed based on XRD patterns and they can be indexed successfully in a hexagonal symmetry with the *P6*_3_*/mmc* space group.^47^ A remarkable shift in XRD patterns toward lower angles with an increasing Nb content from 25 to 75% was observed (Figure 2a). That was expected because of the larger atomic radius of Nb compared to Ti. The XRD patterns of the corresponding MXenes (obtained after acid etching of MAX phase powders) displayed intense (002) reflections with a significant shift toward lower angles, indicating the enlargement of interlayer spacing due to surface functionalization and extraction of the Al layer (Figure 2b).^18^ The splitting of the (002) reflection might be due to the presence of water molecules intercalated between the MXene layers.^48^

Further, the elemental composition of the MXenes was observed by EDS analysis, where stoichiometric Ti:Nb atomic ratios of 1.52:0.48, 1.04:0.96, and 0.55:1.45 were observed for (Ti_0.75_Nb_0.25_)_2_CT_*x*_, (Ti_0.5_Nb_0.5_)_2_CT_*x*_, and (Ti_0.25_Nb_0.75_)_2_CT_*x*_, respectively, with a nearly total removal of Al (Table S1). The corresponding oxidized MXenes are termed TiNbO_*x*_-1:3, TiNbO_*x*_-1:1, and TiNbO_*x*_-3:1 (depending on the initial nominal Ti:Nb ratio in the parent MXenes) in further discussions, where the observed characteristics are linked to the photocatalytic activity of different TiNbO_*x*_ powders.

**Figure 3:**
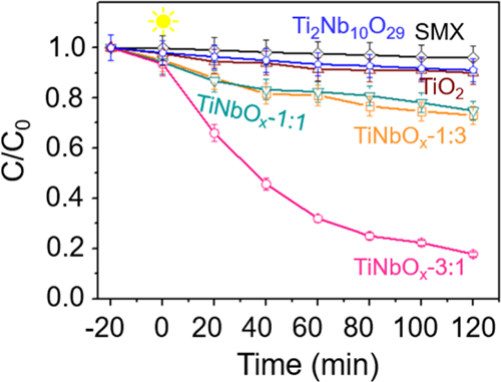
Degradation curves of SMX under UVA in the presence of TiNbO_*x*_ powders for 2 h.

Figure 3 summarizes the performance of three TiNbO_*x*_ powders in the degradation of SMX. For comparison purposes, the photocatalytic activity of TiO_2_ and Ti_2_Nb_10_O_29_ obtained by the oxidation of pristine binary Ti_2_CT_*x*_ and Nb-sustituted quaternary Nb_3.5_Ti_0.5_C_3_T_*x*_, respectively, and the direct photolysis of SMX is also presented in Figure 3. The TiO_2_- and Ti_2_Nb_10_O_29_-derived MXene can degrade up to 10%, while the TiNbO_*x*_ powders exhibited a much higher photocatalytic activity, especially TiNbO_*x*_-3:1, with 83% SMX degradation after 2 h under UVA light. The remarkable photocatalytic behavior of the ternary oxide nanoheterostructures derived from (Ti_*y*_, Nb_1–*y*_)_2_CT_*x*_ MXene is as follows: TiNbO_*x*_-3:1 > TiNbO_*x*_-1:1 > TiNbO_*x*_:1–3 > TiO_2_. Among the three oxide nanoheterostructures, the reusability behavior of the best sample, i.e., TiNbO_*x*_-3:1, was examined through five consecutive cycles, and the photocatalytic efficiency in the SMX degradation slightly decreased from 83 to 72% (Figure S1), thus suggesting that it could be further developed in wastewater treatment plants.

### Relationship between TiNbO_*x*_ Properties and Their Photocatalytic Activity

The observed trend of TiNbO_*x*_ photocatalysts in SMX degradation depends on several factors such as the crystalline phase composition, morphology, surface properties, and electronic band structure. The photocatalytic activities are discussed based on these factors to provide a detailed relationship between these properties.

### Role of Crystalline Phase Composition and Morphology

**Figure 4:**
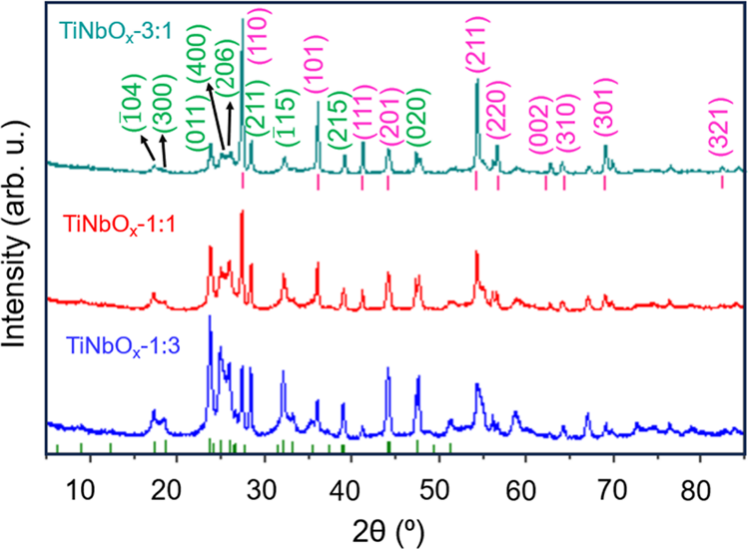
XRD patterns of the complete oxidation of Nb-substituted binary MXenes in air. The reflection marked in pink represents rutile TiO_2_ (PDF#00–001–1292) and that in green corresponds to monoclinic Ti_2_Nb_10_O_29_ (PDF#00–040–0039).

The thermal treatment of MXenes in air resulted in their oxidation and formation of TiNbO_*x*_ (Figures 4a and S2a). Based on reported literature, the oxidation of MXenes such as Ti_3_C_2_T_*x*_ and Ti_2_CT_*x*_ is accompanied by the evolution of CO and CO_2_,^23,49^ which positively impacts the porosity of the oxidized material, thus being beneficial for its photocatalytic properties.^50^ The XRD patterns of TiNbO_*x*_ revealed the formation of rutile TiO_2_ and monoclinic Ti_2_Nb_10_O_29_ (Figure 4a). The reflections observed at the following 2θ values: 23.28, 25.05, 26.07, 27.43, and 28.46° exhibited a shift toward lower angles with an increasing Nb content, attributable to the larger ionic radius of Nb than Ti (Figure 4b). A detailed study of the oxidized MXenes was also gathered by performing Le Bail refinements (Figure S2). The lattice dimensions demonstrated an increase in the cell volume of rutile TiO_2_ and monoclinic Ti_2_Nb_10_O_29_ (Table S2) that can be ascribed to the insertion of Nb into the crystal system of the MXene precursor. Moreover, the inferences of Le Bail refinements and the relative intensity ratio method (by considering w/w %, Figure S2) suggested that the major phase was rutile TiO_2_ (82.34%) along with Ti_2_Nb_10_O_29_ (17.66%) for TiNbO_*x*_-3:1, while Ti_2_Nb_10_O_29_ (62.15%) was predominant in TiNbO_*x*_-1:3 with the competing phase of rutile TiO_2_ (37.85%). In TiNbO_*x*_-1:1, 60.89% TiO_2_ and 39.11% Ti_2_Nb_10_O_29_ were estimated. The Raman spectra of TiNbO_*x*_-3:1, TiNbO_*x*_-1:1, and TiNbO_*x*_-1:3 displayed characteristics bands of Ti_2_Nb_10_O_29_ at 998 and 896 cm^–1^ that correspond to stretching vibrations of the NbO_6_ octahedron, while the bands observed at 549 and 641 cm^–1^ could be assigned to the metal–O stretching vibrations of the TiO_6_ octahedron (Figure S3).^51^ In addition, the intense band depicted at 265 cm^–1^ indicated symmetric and antisymmetric bending vibrations of O–Ti–O and O–Nb–O bridge bonds.^51^ The intense bands observed at 617, 445, and 167 cm^–1^ are the fingerprints of rutile TiO_2_.^52^ Thus, Raman spectra inferences are consistent with the XRD results. The structure of TiNbO_*x*_ samples was examined using FTIR spectroscopy (Figure S4). Two absorptions occurring at 503 and 915 cm^–1^ can be assigned to the stretching vibrations of the terminal Nb–O bond and the bridging Nb–O–Nb bond.^35,53^ The peaks observed at 665 and ∼793 cm^–1^ are the fingerprints of Ti–O–Ti and Nb–O–Nb bridging bonds.^53^ Based on these structural analyses, it can be suggested that the photocatalytic activity of the three samples is due to the presence of transition metal oxides that are known photocatalysts, especially TiO_2_, since the composite has the highest ratio in Ti, leading to the highest efficiency in the photocatalytic degradation of SMX.^54^ However, TiO_2_ obtained from the oxidation of Ti_2_CT_*x*_ MXene exhibited the lowest photocatalytic activity, thus suggesting that the composite formed an efficient heterojunction.

**Figure 5:**
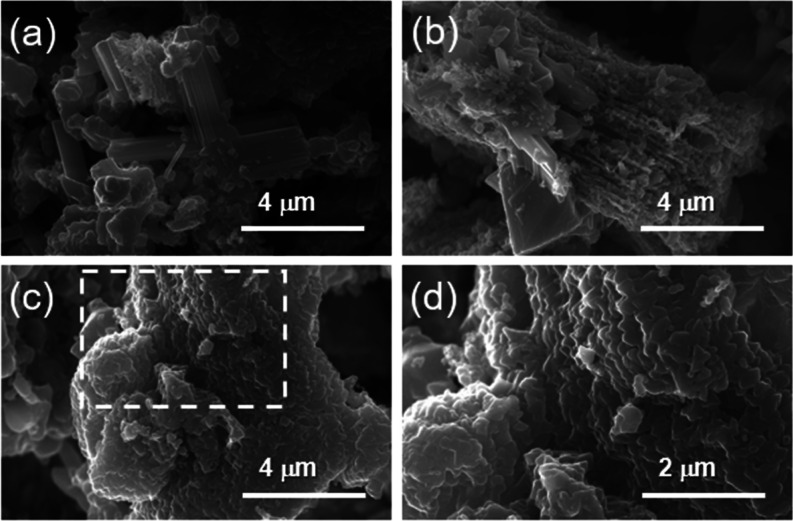
SEM images of (a) TiNbO_*x*_-1:3, (b) TiNbO_*x*_-1:1, (c) TiNbO_*x*_-3:1, and (d) the zoomed region of TiNbO_*x*_-3:1.

The multilayered structure of oxidized MXene seems to be intact after oxidation, and this phenomenon might arise due to insufficient oxidation (Figure 5).^29^ Indeed, during the formation and stabilization of TiO_2_ and Ti_2_Nb_10_O_29_, there might be a surface functionalization with carbon moieties, facilitating the conservation of the layered arrangements.^29^Table S3 confirms the unchanged stoichiometry between Ti:Nb in MXene precursors and TiNbO_*x*_, with significant remaining carbon based on EDS analysis.

**Figure 6:**
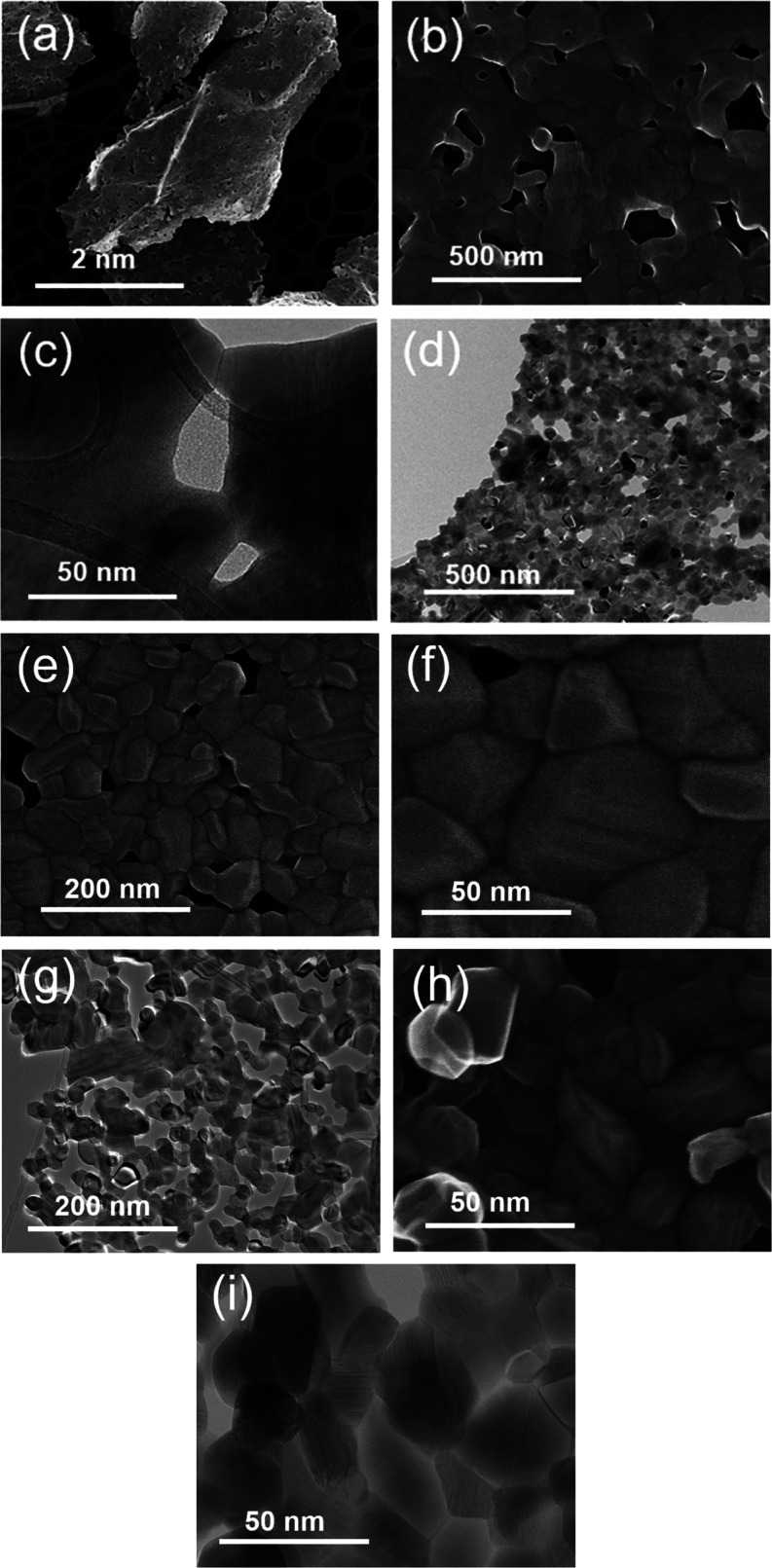
(a, b, e, f, h) SEI STEM and (c, d, g, i) BF STEM images of sheetlike objects at different magnifications for (a–c) TiNbO_*x*_-1:3, (d–f) TiNbO_*x*_-1:1, and (g–i) TiNbO_*x*_-3:1 samples.

The TEM results for TiNbO_*x*_ can provide crucial information in relation to the photocatalytic activity (Figures 6 and S5–S7). In the case of TiNbO_*x*_-1:3, a mesoporous nanosheet-like structure with sizes up to 10 μm is observed, and these nanosheets originate by the interconnection of nanoparticles’ sizes from 50–150 nm in a porous network (Figure 6a).

A better view of the fusion of the nanosized crystallites in nanosheets and in an interconnected fashion can be seen in Figure 6b,c. Similarly, TiNbO_*x*_-1:1 consists of mesoporous nanosheets but with densely packed crystallites with sizes in the range of 40–85 nm and a high porosity (Figure 6d–f). Concerning TiNbO_*x*_-3:1, the mesoporous nanosheet-like structure with densely packed particles is also observed with smaller particle sizes (between 40 and 70 nm) and a larger porosity (Figure 6g–i). We believe that the larger porosity in the TiNbO_*x*_-3:1 sheet could be related to the breakdown of the delaminated nanolayers into smaller aggregates (Figure 6g).

HRTEM imaging and EDS mapping of Ti, Nb, and O elements showed that sheets in TiNbO_*x*_-3:1 and TiNbO_*x*_-1:1 consist of a TiO_2_ and Ti_2_Nb_10_O_29_ mixture (Figures S6 and S7), while sheets in the TiNbO_*x*_-1:3 sample consist majorly of the Ti_2_Nb_10_O_29_ phase and exhibit a uniform distribution of Ti and Nb (Figure S5). From the evaluation of the fast Fourier transform (FFT) pattern shown in Figure S5d, it was determined that the Ti_2_Nb_10_O_29_ crystallite was oriented along the [21̅1] direction, but the crystallite exhibiting the [010] zone axis was also revealed, as shown in Figure S4f. EDS maps of Ti and Nb obtained from TiNbO_*x*_-1:1 and TiNbO_*x*_-3:1 samples revealed an inhomogeneous distribution of Ti and Nb elements in nanosheets (Figures S6a and S7a), which indicates that TiO_2_ and Ti_2_Nb_10_O_29_ nanograins are organized into sheetlike structures. While TiO_2_ nanocrystals significantly prevailed over Ti_2_Nb_10_O_29_ in sample TiNbO_*x*_-3:1, the proportion of TiO_2_ and Ti_2_Nb_10_O_29_ approximately coincided with a ratio of 40:60 in sample TiNbo_*x*_-1:1 (Figure S6a). The presence of rutile and Ti_2_Nb_10_O_29_ nanoparticles in the heterojunction sheetlike material was also accounted for from their HRTEM and respective FFT patterns (Figure S6c–f). Figure 5c exhibits the BF STEM image of a TiO_2_ rutile crystal (dark) viewed along the [010] direction surrounded by Ti_2_Nb_10_O_29_ single crystals, thus confirming the heterojunction formation in TiNbO_*x*_. The TiO_2_/Ti_2_Nb_10_O_29_ nanostructure emerges where the coexistence of TiO_2_ (marked R) and Ti_2_Nb_10_O_29_ grains is inferred from the HRTEM method (Figure S6b). The detailed HRTEM image of Ti_2_Nb_10_O_29_ with the respective FFT pattern exhibiting a zone axis of [21̅1] is presented in Figure S6e,f. It explains why TiNbO_*x*_ is more efficient than TiO_2_ in the photocatalytic degradation of SMX (Figure 3).

Among all three samples, TiNbO_*x*_-3:1 possesses a higher porosity and smaller crystallite sizes, resulting in increased surface-active sites, favoring its higher photocatalytic activity. The HRTEM images of the three TiNbO_*x*_, along with the corresponding FFT or SAED patterns, confirmed the presence of TiO_2_ and Ti_2_Nb_10_O_29_ (Figures S5–S7), thus supporting the XRD data. As seen from Figures S6c,d and S7c,d, rutile crystals in nanosheets of TiNbO_*x*_-1:1 and TiNbO_*x*_-3:1 samples are well faceted. By evaluating the FFT pattern, as shown in Figure S7d, gained from Figure S7c, a rutile single crystal is enclosed by combining the {110} and {101} type planes, which are prismatic and pyramidal facets, respectively. The same result is also shown in Figure S6c,d. The presence of oxidation preferred faces of the {101} type and the reduction preferred faces of the {110} type, enclosing the nanocrystals in the TiNbO_*x*_-3:1 sample with the highest proportion of rutile TiO_2_ can support the high photocatalytic activity of TiNbO_*x*_-3:1.^55^ Furthermore, the appearance of nanotwins and voids in rutile nanocrystals of TiNbO_*x*_-3:1 (Figure S7e,f) is responsible for elevating free charge transfer as well as facilitates the separation of charge carriers, which further may help in boosting the photocatalytic activity.^56^ These morphological features may support the photocatalytic behavior since TiNbO_*x*_-3:1 is the most efficient sample in the degradation of SMX.

BET analysis of the samples is provided in Table 1. The data predominantly showed the macromesoporous nature of the TiNbO_*x*_ samples. However, micropores in the TiNbO_*x*_-3:1 sample suggested its multiscale porosity. Indeed, materials with a multiscale porosity are highly desirable as they can improve the overall photocatalytic efficiency by enhancing the mass transfer of molecules into solids and the light utilization efficiency.^57^ TiNbO_*x*_-3:1 has a significantly higher surface area, further supporting its higher photocatalytic activity for SMX degradation.

**Table 1:** Characteristic Parameters for TiNbO_*x*_ Obtained from BET Analysis^a^

sample identification	*a* [m^2^/g]	*b* [cm^3^/g]	*c* [cm^3^/g]	*d* [cm^3^/g]	*e* [cm^3^/g]	*f*
TiNbO_*x*_-1:3	^+^0.5	^+^0.11	0.011	0	0.025	few ME, no MI, with multiple MA
TiNbO_*x*_-1:1	^+^0.3	^+^0.06	0.007	0	0.010	few ME, no MI, with multiple MA
TiNbO_*x*_-3:1	^+^4	^+^1.0	0.014	0.001	0.021	little ME, less MI, with more MA

a *a*: specific surface area according to BET,^58^ marked +; *b*: volume of the adsorbed monomolecular layer; *c*: cumulative pore volume according to Gurvich; *d*: cumulative volume of micropores (MIs) according to Horvath–Kawazoe; *e*: cumulative volume of the mesopore (ME) and macropore (MA) according to the BJH method; and *f*: nature of the material.

### Role of Surface Chemistry

**Figure 7:**
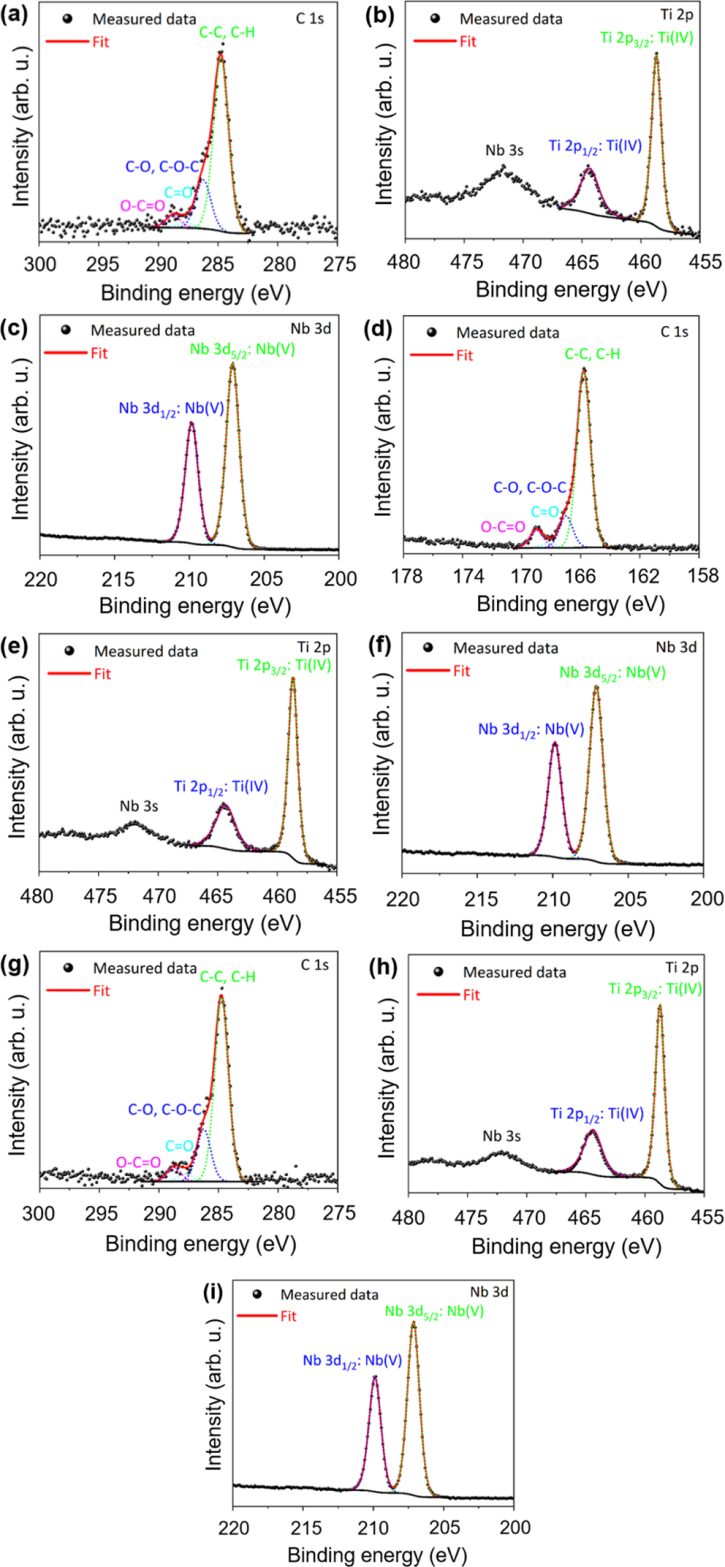
XPS C 1s, Ti 2p, and Nb 3d core-level spectra of (a–c) TiNbO_*x*_-1:3, (d–f) TiNbO_*x*_-1:1, and (g–i) TiNbO_*x*_-3:1. The spectra are referenced using the C–C/C–H component at 284.8 eV.

The materials’ photocatalytic efficiency is generally intricately linked to their surface chemistry. The wide spectra (Figure S8) documented the presence of O, C, Nb, and Ti on the surface of the TiNbO_*x*_ samples. To further elucidate the surface chemistry, we thoroughly analyzed the core-level spectra of C 1s, Ti 2p, and Nb 3d orbitals, as presented in Figure 7. Notably, XPS detects the signal from a 2–10 nm depth below the surface. The C 1s spectra of the samples (Figure 7a,d,g) consist of components at positions of 284.8, 286.3, 287.8, and 288.8 eV of binding energies ascribed to C–C (or C–H), C–O (or C–O–C), C=O, and O–C=O species, corresponding to adventitious carbon contaminants.^43^ The relative share of the components in the C 1s spectra due to the exposure to an ambient atmosphere was very similar among the TiNbO_*x*_ samples (Table S4). Therefore, the formation of carbon species on the surface was not affected by the stoichiometry of oxidized MXenes. It is known that the hydrophilic groups on the surface (C–O, C=O, O–C=O) of photocatalysts can enhance the photocatalytic activity due to the improved interaction with organic pollutants.^59^

Nevertheless, the higher photocatalytic performance of the TiNbO_*x*_-3:1 sample compared to the other counterparts cannot be explained by these detected hydrophilic groups since their ratios are very similar for them (Table S4). The Ti 2p spectra (Figure 7b,e,h) comprised the doublet lines Ti 2p_3/2_ and Ti 2p_1/2_ at binding energies of 458.7 and 464.4 eV, respectively. The doublet line splitting of 5.7 eV and the positions of the components reflect the presence of Ti ions in the +IV oxidation state. This supports the structure of Ti_2_Nb_10_O_29_ and the presence of TiO_2_,^44^ detected by XRD within the bulk area of the samples. The Nb 3d core-level spectra of the TiNbO_*x*_ samples (Figure 7c,f,i) show the doublet lines Nb 3d_5/2_ and Nb 3d_3/2_ at binding energies of 207.1 and 209.8 eV. The deconvolution of the spectra to the corresponding components reflects the presence of Nb in the +V oxidation state, corresponding to Ti_2_Nb_10_O_29_.^35,42^

### Role of the Electronic Band Structure

**Figure 8:**
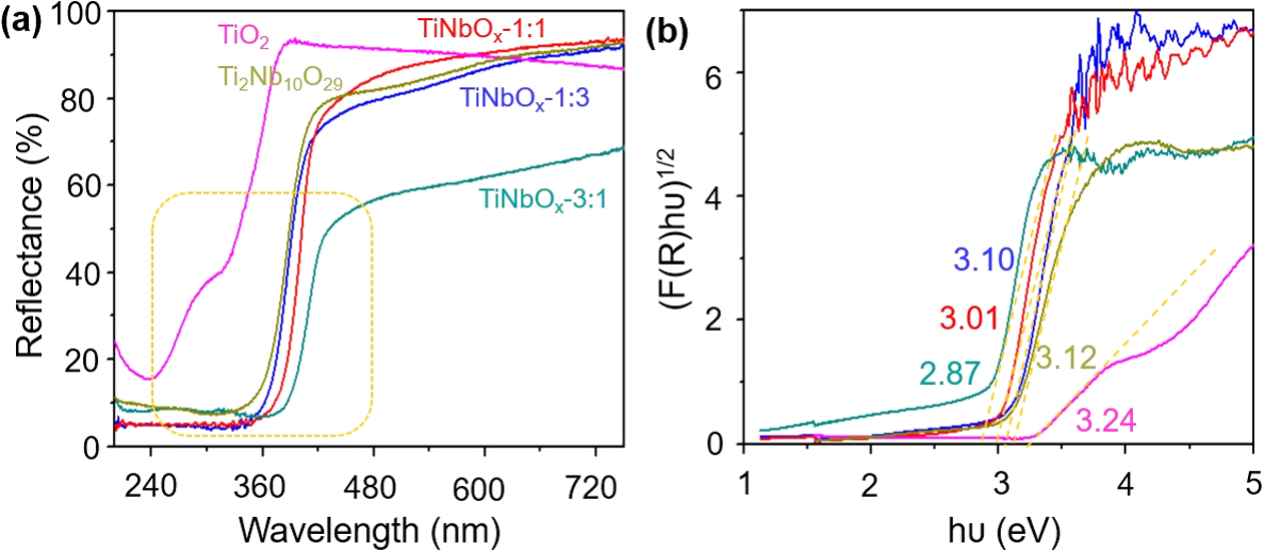
(a) UV–visible DRS of TiO_2_, Ti_2_Nb_10_O_29_, and TiNbO_*x*_ samples and their corresponding (b) band gap plots.

Figure 8 displays the UV–vis DRS spectra of the TiNbO_*x*_ samples. A red shift was observed with a decreasing Nb content in TiNbO_*x*_, i.e., UV to the visible region (Figure 8a). The optical band gap (*E*_g_) energy decreases as the Nb content decreases. By assuming an indirect energy band gap for plotting the Tauc’s plots, the estimated *E*_g_ is as follows: TiNbO_*x*_-1:3 > TiNbO_*x*_-1:1 > TiNbO_*x*_-3:1 with the corresponding values of 3.10, 3.01, and 2.87 eV (Figure 8b).^60^ Based on XPS studies, we detected a similar surface chemistry in samples; therefore, we assume that the high photocatalytic behavior of TiNbO_*x*_-3:1 primarily can be attributed to bulk properties rather than surface characteristics. TiO_2_ is well known to exhibit a higher photocatalytic activity when enriched with bulk or volume defects instead of surface defects.^61^ Based on TEM analysis (Figure S7f), the exceptional behavior of TiNbO_*x*_-3:1 may originate from the presence of volume defects as voids within the TiO_2_ nanocrystal.^62^ Additionally, a narrower energy band gap will lead to a higher utilization of solar light, i.e., in both the UVA and visible regions.

**Figure 9:**
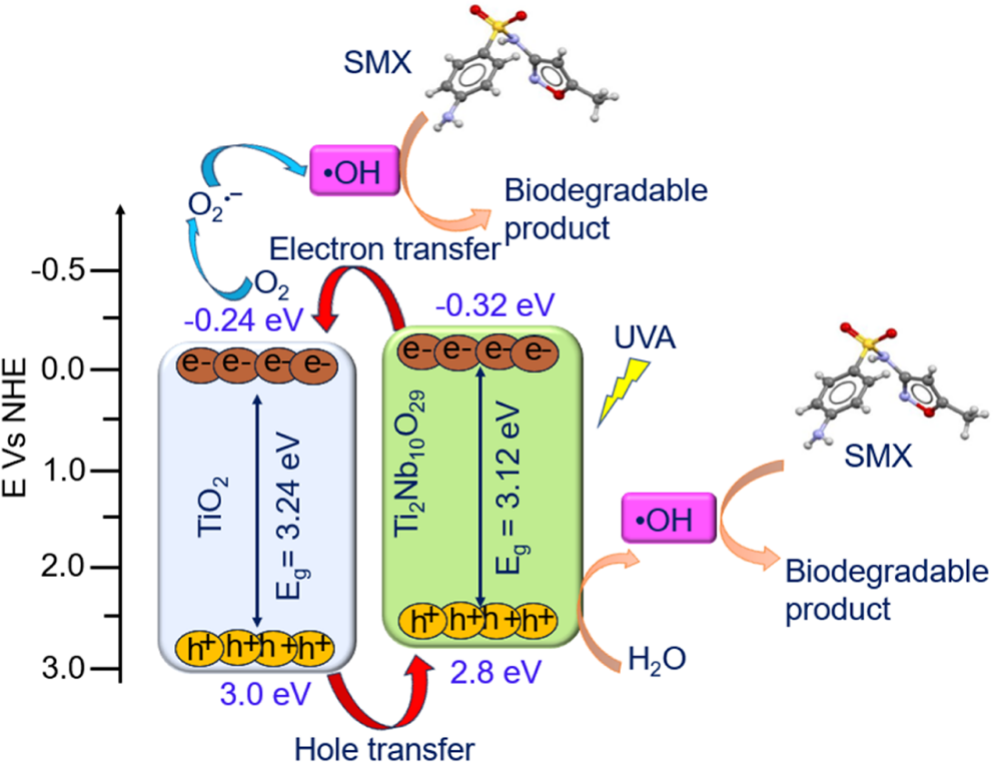
Proposed mechanism of SMX photocatalytic degradation by using TiNbO_*x*_.

The TiNbO_*x*_ samples have been found to exhibit a reduced band gap compared to pure rutile TiO_2_ (3.24 eV) and monoclinic Ti_2_Nb_10_O_29_ (3.12 eV) phases derived by the oxidation of Ti_2_CT_*x*_ and Nb_3.5_Ti_0.5_C_3_T_*x*_ and it might be due to structural defects brought by the synthesis method as explained above. In addition, the valence band maximum of TiNbOx analyzed by UV photoelectron spectroscopy has been calculated at 2.90 eV (Figure S9).^63^ Therefore, the electronic band structure of TiNbO_*x*_ can be reasonably estimated as a type-II heterojunction (Figure 9).^64−66^ Such a heterojunction will favor the charge carrier’s separation, where photogenerated electrons are accumulated in TiO_2_ and photogenerated holes in Ti_2_Nb_10_O_29_ under UVA illumination (Figure 9). This is one main reason why TiNbO_*x*_ exhibited a higher photocatalytic efficiency than TiO_2_. Among the TiNbO_*x*_ samples, the highest photocatalytic activity is observed for TiNbO_*x*_-3:1, probably because rutile TiO_2_ (the predominant phase in this sample) is more photoactive than Ti_2_Nb_10_O_29_.^67^

### Proposed Photocatalytic Mechanism

The photocatalytic processes using TiO_2_-based materials are usually accompanied by the generation of reactive oxygen species (ROS) such as ^•^OH and O_2_^•–^, from which the hydroxyl radical is the predominant one.^68,69^ Indeed, superoxide anion radicals can be converted to H_2_O_2_ and ultimately into ^•^OH.^70^ To detect ^•^OH, coumarin has been used as a probe molecule since it forms 7-hydroxycoumarin (7OH–C) (with a yield of 4.6%),^42^ which can be easily detectable by using a fluorescence spectrophotometer. The amount of ^•^OH formation for TiNbO_*x*_-3:1 was twice and thrice that of TiNbO_*x*_-1:1 and TiNbO_*x*_-1:3 (Figure S10), which supports its higher photocatalytic activity in the degradation of SMX.

Further, *t*-butanol was employed as a selective scavenger for ^•^OH during photocatalytic measurements, and the results showed a complete quench of the photocatalytic degradation of SMX (Figure S10), which implied that ^•^OH is the predominant ROS involved in the degradation reactions. Based on the estimated electronic band structure of TiNbO_*x*_, the mechanism of SMX degradation is proposed. The lower photocatalytic activities of TiNbO_*x*_-1:1 and TiNbO_*x*_-1:3 in the SMX degradation can be correlated with the concentration of TiO_2_. As per the estimated electronic band structure, under UVA irradiation, e̅ is accumulated in TiO_2_ and h^+^ in Ti_2_Nb_10_O_29_ by the following eq 1. The higher photocatalytic activity of TiNbO_*x*_-3:1 is accompanied by the generation of a large number of ^•^OH, by the interaction of h^+^ with H_2_O/OH̅ and e̅ with the adsorbed O_2_ molecule on the surface of the catalyst by the following eqs 2–5.^71^
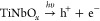
1
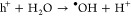
2
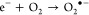
3
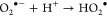
4
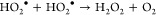
5
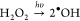
6Notably, pure TiO_2_ and Ti_2_Nb_10_O_29_ derived from the oxidation of MXenes exhibited the weakest activity in SMX degradation. It confirmed the importance of Nb substitution in binary MXenes, which are precursors to preparing innovative photocatalysts. Compared to other reported studies, which are mainly focused on dye degradation (Table S5), the current work highlights the significance of our findings since innovative nanoheterostructures composed of Ti and Nb oxides are efficient photocatalysts for the degradation of SMX, which is considered a PAC. Since all of the TiNbO_*x*_ samples are composed of the type-II heterojunction with a similar surface chemistry and layered morphology derived from their MXene precursor, the higher superior photocatalytic activity of TiNbOx-3:1 is attributed to its higher porosity and a higher Ti:Nb ratio in TiO_2_, thus leading to an efficient production of ^•^OH.

## Conclusions

For the first time, innovative oxide nanoheterostructures were prepared by oxidizing Ti–Nb MXenes, and their photocatalytic performance in the degradation of SMX under UVA light was evaluated. Among the three tested TiNbO_*x*_ samples composed of TiO_2_ and Ti_2_Nb_10_O_29_, TiNbO_*x*_-3:1 exhibited the best degradation performance. The complex interplay between the structural, morphological, and electronic properties of TiNbO_*x*_ suggested that a higher Ti:Nb ratio, smaller particle size, and larger specific surface area were the key factors contributing to its superior photocatalytic performance. This study underscores the complex influence of the structural, morphological, and electronic properties on the photocatalytic properties of TiNbO_*x*_. Moreover, the proposed degradation mechanism invoves ^•^OH as the primary ROS, produced in significantly high amounts in the presence of TiNbO_*x*_-3:1. The primary conclusion emerging from the present work illustrates TiNbO_*x*_-3:1 as an efficient oxide nanoheterostructure photocatalyst, which can be explored further in wastewater treatment.

## Supporting Information Available

Recyclability performance of TiNbO*_x_*-3:1; Le Bail refinements; Raman spectra; FTIR spectra; TEM images; XPS wide spectra; UPS spectra; relative share of the C 1s components of TiNbOx samples; literature survey table (PDF)
