# Supplementary material for: MXene-Derived Oxide Nanoheterostructures for Photocatalytic Sulfamethoxazole Degradation
Source: ACS Appl Nano Mater. 2024 Jul 18;7(14):16506–15. doi: 10.1021/acsanm.4c02523 (PMC11287779; doi:10.1021/acsanm.4c02523)
Supplement: Supplementary file 1 — an4c02523_si_001.pdf [file an4c02523_si_001.pdf]

Supplementary Information file

MXene-Derived Oxide Nano-Heterostructures for Photocatalytic Sulfamethoxazole Degradation

Shalu Atri,[a,b*] Elham Loni,[b] Frantisek Zazimal,[c] Karol Hensel,[d] Maria Caplovicova,[e] Gustav Plesch,[a] Xin Lu,[b] Rajamani Nagarajan,[f] Michael Naguib,[b,g*] Olivier Monfort[a*]

[a] Department of Inorganic Chemistry, Faculty of Natural Sciences, Comenius University, Ilkovicova 6, Mlynska dolina, 84215 Bratislava, Slovakia

[b] Department of Physics and Engineering Physics, Tulane University, New Orleans, Louisiana, United States of America

[c] Department of Plasma Physics and Technology, Faculty of Science Masaryk University, Masaryk University, Kotlarska 267/2, 611 37 Brno, Czechia

[d] Division of Environmental Physics, Faculty of Mathematics Physics and Informatics, Comenius University, Mlynska dolina, 84248 Bratislava, Slovakia

[e] STU Center for Nanodiagnostics, Faculty of Materials Science and Technology in Trnava, Slovak Technical University, Vazovova 5, 81243 Bratislava, Slovakia.

[f] Materials Chemistry Group, Department of Chemistry, University of Delhi, Delhi- 110007, India

[g] Department of Chemistry, Tulane University, New Orleans, Louisiana, United States of America

*Corresponding authors: shalu1@uniba.sk (SA); naguib@tulane.edu (MN); monfort1@uniba.sk (OM)

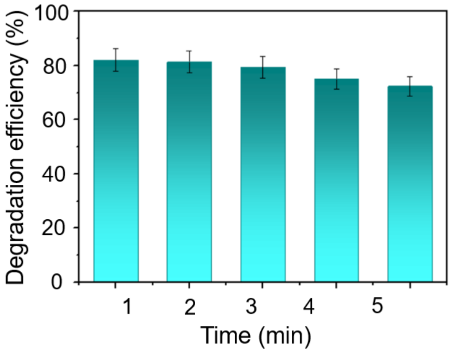

Figure S1 Recyclability performance of $TiNbO_x$-3:1, up to five consecutive cycles.

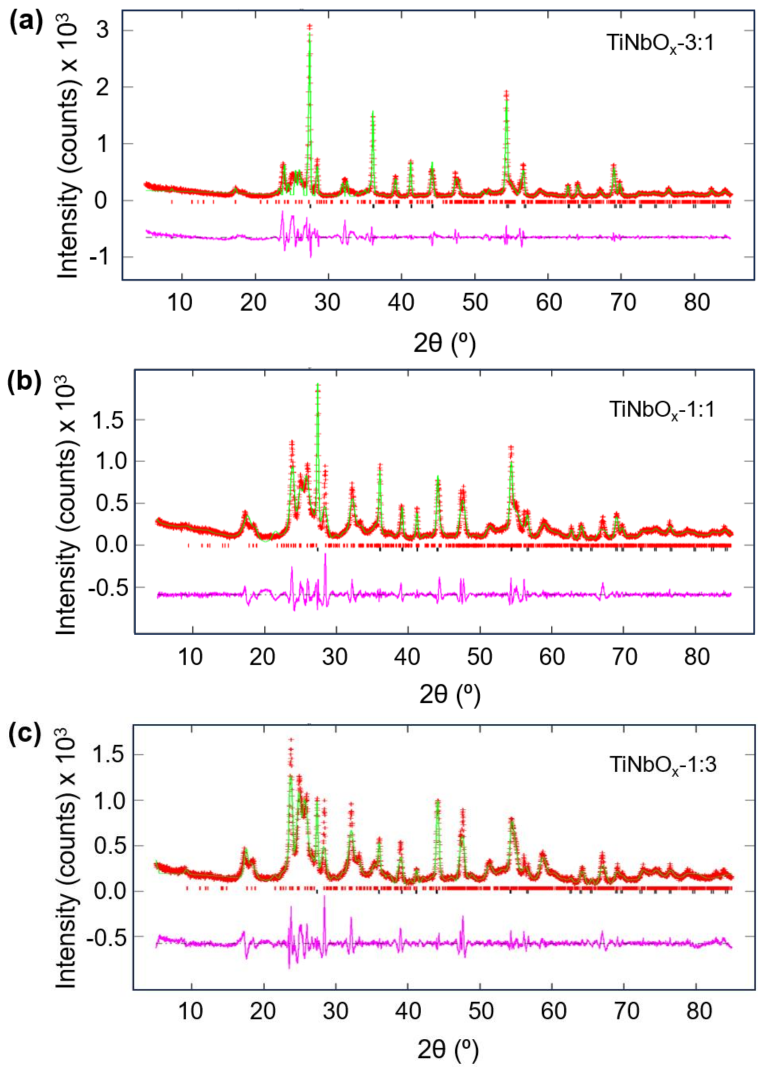

Figure S2 Lattice refinements of the PXRD patterns of the (a) $TiNbO_x$-3:1, (b) $TiNbO_x$-1:1, and (c) $TiNbO_x$-1:3 by the Le Bail method. The red, green, and pink lines are for the experimental data, the calculated, and the difference profiles, respectively.

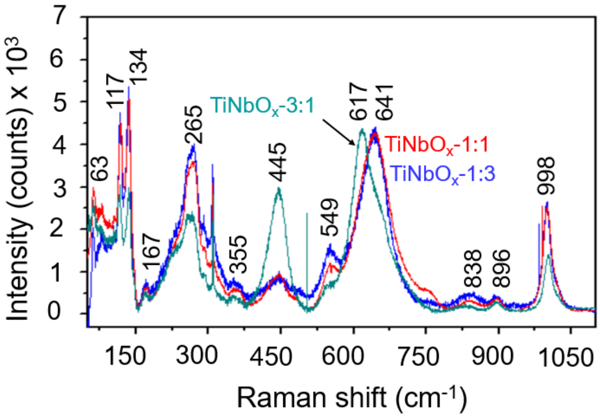

Figure S3 Raman spectra of $TiNbO_x$-1:3, $TiNbO_x$-1:1, $TiNbO_x$-3:1.

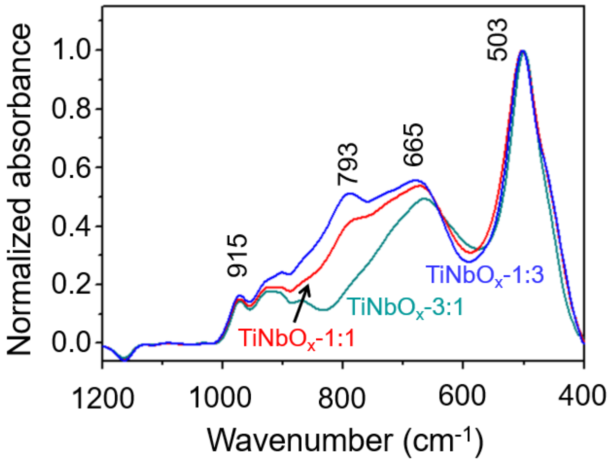

Figure S4 FTIR spectra of $TiNbO_x$-1:3, $TiNbO_x$-1:1, $TiNbO_x$-3:1.

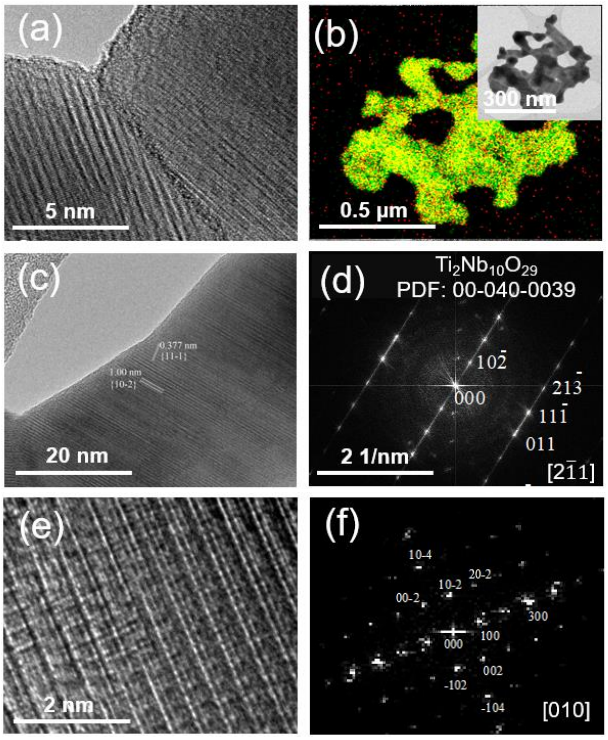

Figure S5 $TiNbO_x$-1:3 sample, (a) Detail of interface of coalesced $Ti_2Nb_{10}O_{29}$ nanoparticles. (b) EDS mapping of Ti and Nb distribution in nanosheet. TiK and NbL X-ray maps are overlayed. Inset is BF STEM image of the same aggregate. HRTEM image (c) and relevant FFT pattern (d) of $Ti_2Nb_{10}O_{29}$ crystallite oriented along $[1\bar{1}1]$ direction. HRTEM image (e) and relevant FFT pattern(f) of $Ti_2Nb_{10}O_{29}$ crystallite oriented along [010] direction.

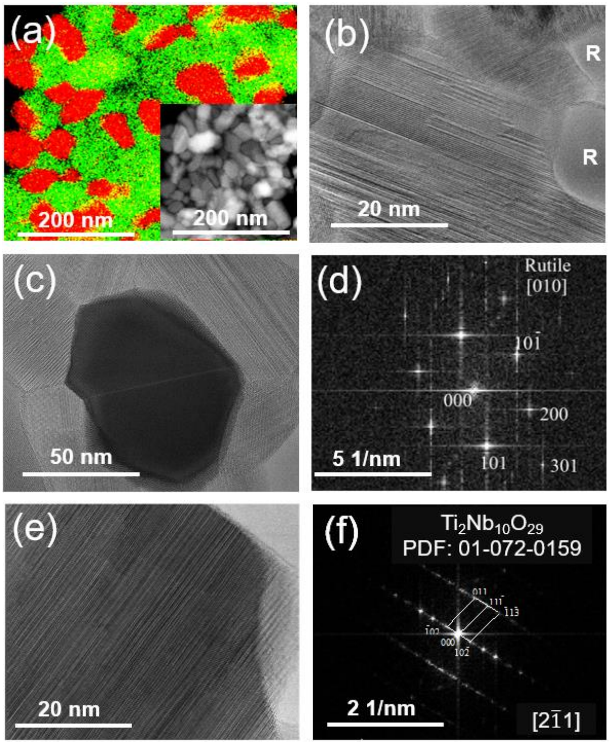

Figure S6 TiNbO$_x$-1:1 sample (a) EDS mapping of TiK and NbL distribution in nanosheet. overlayed TiK and NbL x-ray maps. Inset is the HAADF STEM image of the same aggregate. (b) HRTEM image of nanosheet showing $Ti_2Nb_{10}O_{29}$ (in the middle) and TiO_2 grains (R). (c) HRTEM detail of rutile TiO_2 crystallite (dark) with zone axis [010] and relevant FFT pattern in (d). (e) HRTEM of $Ti_2Nb_{10}O_{29}$ crystallite with zone axis [$2\bar{1}1$]. (f) FFT pattern acquired from (e).

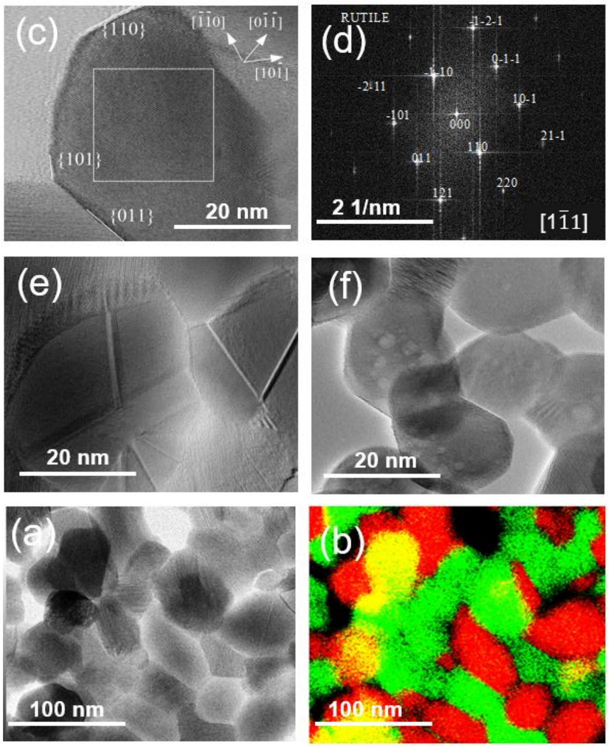

Figure S7 (a) and (b) BF STEM image of the nanosheet and EDS mapping (TiK and NbL maps overlayed) of TiNbOx-3:1 sample, respectively. (c) BF STEM image of TiO_2 rutile single crystal oriented along $[1\bar{1}1]$ direction along with surface planes marked. (d) Relevant FFT pattern, (e) nano twins in rutile nanocrystals and (f) voids in rutile.

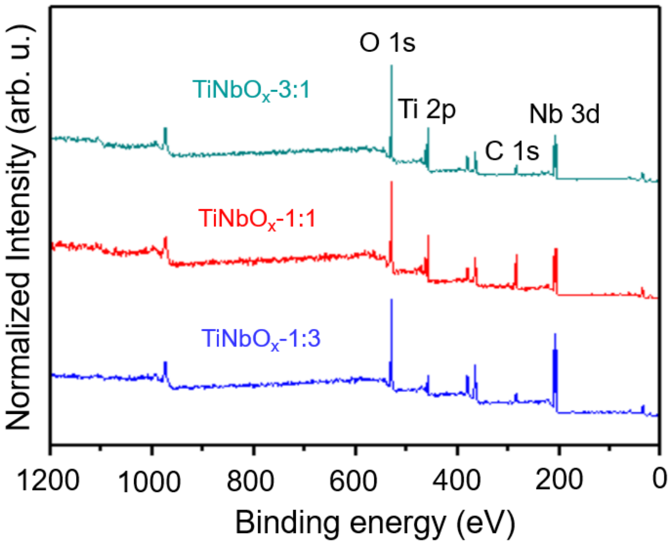

Figure S8 The wide XPS spectra of $TiNbO_x$ samples.

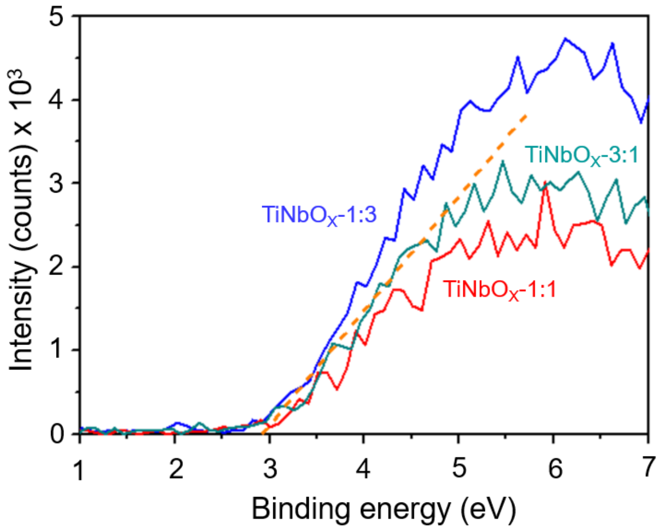

Figure S9 The ultraviolet photoelectron spectroscopy (UPS) spectra of $TiNbO_x$ samples.

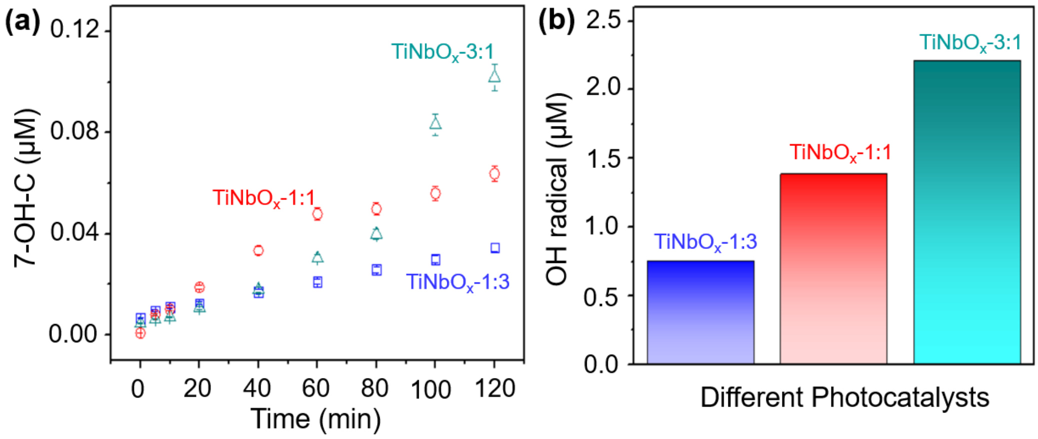

Figure S10 (a) 7OH-C production using coumarin as a probe molecule under UVA using $TiNbO_x$ samples and (b) the corresponding estimation of hydroxyl radicals over a period of 2 h.

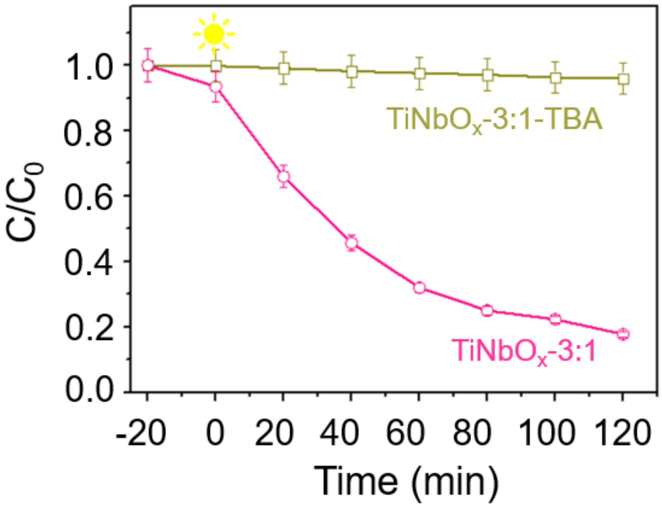

Figure S10 Degradation performance of $TiNbO_x$-3:1photocatalyst in the presence of t-butanol as a scavenger.

Supplementary Information file

Table S1 Elemental composition in at% of the Nb-substituted binary MXenes obtained by EDS.

Element	$(Ti_{0.25}Nb_{0.75})_2CT_x$	$(Ti_{0.50}Nb_{0.50})_2CT_x$	$(Ti_{0.75}Nb_{0.25})_2CT_x$
**Ti**	7.93±5.88	15.18±8.96	18.06±2.83
**Nb**	18.63±7.19	13.25±6.45	5.52±0.55
**O**	17.06±4.53	17.89±6.23	24.05±0.73
**C**	51.14±10.12	42.60±8.47	36.64±2.34
**F**	3.72±1.86	5.58±2.32	11.35±0.71
**Cl**	-	4.75±1.39	4.04±0.45
**Al**	1.52±1.09	0.77±0.29	0.34±0.21

Table S2 Lattice dimensions of the $TiNbO_x$ samples obtained after Le Bail refinements.

	$TiNbO_x$-1:3	$TiNbO_x$-1:1	$TiNbO_x$-3:1
**TiO_2 (Tetragonal, *$P4_2/mnm$*)**	a = 4.596 (5) Å<br>c = 2.965 (7) Å<br>Cell volume=62.67(17) $Å^3$	a = 4.595 (2) Å<br>c = 2.960 (3) Å<br>Cell volume= 62.52(7) $Å^3$	a = 4.592(2) Å<br>c = 2.958(2) Å<br>Cell volume= 62.41(6) $Å^3$
**$Ti_2Nb_{10}O_{29}$ (Monoclinic, *C2/m*)**	a = 15.886(2)<br>b = 3.847(4)<br>c = 20.737(2)<br>β = 114.677(1)<br>Cell volume=1151.8(1) $Å^3$	a = 15.736(5)<br>b = 3.833(6)<br>c = 20.653(4)<br>β = 114.460(22)<br>Cell volume=1134.0(3) $Å^3$	a = 15.541(4)<br>b = 3.828(6)<br>c = 20.474(3)<br>β = 112.91(5)<br>Cell volume=1122.0(3) $Å^3$
**Goodness of fit**	2.38	2.39	2.57

Table S3 Elemental composition in at% obtained by EDS of $TiNbO_x$-1:3, $TiNbO_x$-1:1, $TiNbO_x$-3:1.

Element	$TiNbO_x$-1:3	$TiNbO_x$-1:1	$TiNbO_x$-3:1
Ti	3.41±0.11	7.36±0.28	13.13±0.98
Nb	8.98±0.15	7.13±0.13	4.19±0.37
O	62.51±0.67	65.66±0.70	68.65±2.28
C	23.82±0.72	18.92±0.48	13.02±3.26
F	0	0.06±0.08	0.15±0.14
Cl	0	0.02±0.01	0.01±0.01
Al	1.26±0.07	0.86±0.03	0.86±0.09

Table S4 The relative share of the C 1s components of $TiNbO_x$ samples.

Sample	C-C (C-H) (%)	C-O (C-O-C) (%)	C=O (%)	O-C=O (%)
$TiNbO_x$-1:3	72.1	20.4	2.4	5.1
$TiNbO_x$-1:1	76.5	13.8	2.0	7.7
$TINbO_x$-3:1	71.4	20.3	3.0	5.3

Table S5 Photocatalytic performance of Ti and Nb oxides photocatalysts in the degradation of organic pollutants in water.

Photocatalyst (dosage)	Pollutant* (conc.)	Irradiation	Reaction time	Degradation efficiency	Ref.
$TiNbO_x$ nano-heterostructure (0.2 g L^{-1})	SMX (50 µM)	UVA	120 min	83%	This work
Nb_2O_5/TiO_2 heterostructure (1.0 g L^{-1})	RhB (10 µM)	UVC	90 min	100 %	[1]
Nb_2O_5/TiO_2 heterojunction (0.3 g L^{-1})	PhEtOH (25 mM)	UV-Vis	360 min	>95%	[2]

TiO_2 supported on Nb_2O_5 (0.3 g L^{-1})	ATR (23.18 µM), DFC (16.88 µM), IC (64.38 µM)	UV	120 min	> 90%	[3]
Nb-TiO_2 nanocomposites (100 mg L^{-1})	PhR (56.43 µM)	UV	110 min	80 %	[4]
2mol% Nb-TiO_2 nanocomposites (100 mg L^{-1})	PhR (56.43 µM)	UV	160 min	94%	[5]
5mol% Nb_2O_5-TiO_2 nanoparticles (170 mg L^{-1})	MB (10 µM)	UV	150 min	100%	[6]
Nb-TiO_2 (1.6 g L^{-1})	Orange-II (57.09 µM)	UVC	120 min	100 %	[7]
3mol% Nb–TiO_2 (1.0 g L^{-1})	MB (62.53 µM), RhB (41.75 µM)	UV	150 min	35% MB; 45% RhB	[8]
$Ti_2Nb_{10}O_{29}$ (1 g L^{-1})	RhB (10.43 µM)	UV	150 min	95 %	[9]
5mol% Nb–TiO_2 (0.25 g L^{-1})	MB (62.53 µM)	Vis	100 min	65%	[10]

*Sulfamethoxazole = SMX; Rhodamine B = RhB; α-phenylethanol = PhEtOH; Atrazine = ATR; Diclofenac = DFC; Indigo carmine = IC; Phenol red = PhR; Methylene Blue = MB

Reference

[1] Ucker, C.L., Riemke, F., Goetzke, V., Moreira, M.L., Raubach, C.W., Longo, E., Cava, S. Facile preparation of Nb_2O_5/TiO_2 heterostructures for photocatalytic application. *Chem. Phys. Impact*, 2022, *4*, 100079.

[2] Yan, J., Wu, G., Guan, N., Li, L. Nb_2O_5/TiO_2 heterojunctions: synthesis strategy and photocatalytic activity. *Appl. Catal. B Environ.* 2014, *152*, 280-288.

[3] de Andrade, F.V., de Lima, G.M., Augusti, R., Coelho, M.G., Assis, Y.P., Machado, I.R. A new material consisting of TiO_2 supported on Nb_2O_5 as photocatalyst for the degradation of organic contaminants in aqueous medium. *J. Environ. Chem. Eng.* 2014, *2*(4), 2352-2358.

[4] Almulhem, N.K., Awada, C., Alnaim, N.M., Al Taisan, N., Alshoaibi, A.A., Shaalan, N.M. Synergistic Effect of the $KBrO_3$ Electron Acceptor on the Photocatalytic Performance of the Nb-TiO_2 Nanocomposite for Polluted Phenol Red Wastewater Treatment. *Crystals* 2022, *12*(12), 1758.

[5] Almulhem, N., Awada, C., Shaalan, N.M. Photocatalytic degradation of phenol red in water on Nb(x)/TiO_2 nanocomposites. *Crystals* 2022, *12*(7), 911.

[6] da Silva, A.L., Muche, D.N., Dey, S., Hotza, D., Castro, R.H. Photocatalytic Nb_2O_5-doped TiO_2 nanoparticles for glazed ceramic tiles. *Ceram. Inter.* 2016, *42*(4), 5113-5122.

[7] Znad, H., Ang, M., Tade, M. Ta/TiO_2-and Nb/TiO_2-mixed oxides as efficient solar photocatalysts: preparation, characterization, and photocatalytic activity. *Int. J. Photoenergy* 2012, *2012*, 548158-1.

[8] da Silva LF, Avansi W, Catto AC, Rodrigues JEFS, Bernardi MIB, Mastelaro VR. The role of Nb addition in TiO_2 nanoparticles: phase transition and photocatalytic properties. *Phys. Status Solidi Appl. Mater. Sci.* 2018, *215*(21), 1-8.

[9] Xie, M., Zhu, H., Fang, M., Huang, Z., Liu, Y.G., Wu, X. Band-gap engineering and comparative investigation of $Ti_2Nb_{10}O_{29}$ photocatalysts obtained by various synthetic routes. *Appl. Surf. Sci.* 2018, *435*, 39-47.

[10] Ferrari-Lima, A.M., Marques, R.G., Gimenes, M.L., Fernandes-Machado, N.R.C. Synthesis, characterisation and photocatalytic activity of N-doped TiO_2-Nb_2O_5 mixed oxides. *Catal. Today* 2015, *254*, 119-128.
